# Unveiling the Role of [18F]FDG PET/CT in NUT Carcinoma: Insights from a Case Series and Literature Review

**DOI:** 10.3390/diagnostics16193155

**Published:** 2026-09-28

**Authors:** Claudia Battisti, Dino Rubini, Anna Giulia Nappi, Cristina Ferrari, Giuseppe Rubini

**Affiliations:** 1 Nuclear Medicine Unit, Interdisciplinary Department of Medicine (DIM), University of Bari “Aldo Moro”, 70124 Bari, Italy; claudiabattisti93@gmail.com (C.B.); anna.giulia.nappi@gmail.com (A.G.N.); giuseppe.rubini@uniba.it (G.R.); 2 Interdisciplinary Department of Medicine, Section of Radiology and Radiation Oncology, University of Bari “Aldo Moro”, 70124 Bari, Italy; rubini.dino@libero.it

**Keywords:** NUT carcinoma, [18F]FDG PET/CT, pediatric and young-adult patients, rare tumor, metabolic imaging

## Abstract

**Background**: NUT carcinoma (NC) is a rare and highly aggressive NUTM1-rearranged malignancy characterized by rapid progression, frequent metastatic dissemination, and poor clinical outcomes. Evidence regarding the role of [18F]FDG PET/CT remains limited and is largely based on isolated case reports and small series. We describe serial [18F]FDG PET/CT findings in three pediatric and young-adult patients with NC and place these observations in the context of a structured narrative review of the available PET literature. **Case Series and Methods**: Three patients with histologically confirmed NC arising from the anterior chest wall, head and neck region, and mediastinal region underwent [18F]FDG PET/CT at baseline and during treatment or follow-up. All primary tumors were FDG-avid, although metabolic activity, disease distribution, treatment-related imaging changes, and clinical outcomes were markedly heterogeneous. Baseline PET/CT depicted FDG-avid primary, nodal, and distant disease; in one patient, skeletal lesions were identified without corresponding CT abnormalities, while in another, an additional skeletal PET finding prompted whole-body MRI evaluation. Serial PET/CT documented longitudinal changes in FDG uptake and disease distribution, without assigning formal metabolic response categories. The patient with localized disease remained alive and in complete clinical remission at 30 months, whereas the two patients with metastatic disease died 11 and 12 months after diagnosis, respectively. Baseline MTV and TLG showed substantial inter-patient variability and were explored only as descriptive quantitative measures of metabolically active disease burden. **Conclusions**: These observations illustrate the potential complementary contribution of [18F]FDG PET/CT to multimodality imaging in NUT carcinoma, particularly for whole-body disease depiction and assessment of longitudinal metabolic changes. MTV and TLG should be regarded as exploratory, method-dependent descriptive measures and are not validated biomarkers in NC. Larger multicenter studies using standardized acquisition, analysis, and longitudinal interpretation methods are required.

## 1. Introduction

Nuclear protein in testis (NUT) carcinoma (NC), previously referred to as NUT midline carcinoma, is a rare, highly aggressive, and poorly differentiated squamous carcinoma defined by its molecular profile. The disease is characterized by chromosomal rearrangements involving the NUTM1 gene, most commonly the t(15;19)(q14;p13.1) translocation resulting in BRD4:NUTM1 fusion, although several alternative fusion partners have also been described [1,2,3,4]. The resulting fusion oncoprotein blocks epithelial-squamous differentiation, maintains the proliferation of immature neoplastic cells, and promotes uncontrolled tumor growth [4,5]. The first case was described by Kubonishi et al. in 1991 in a woman with a presumed thymic carcinoma refractory to chemo- and radiotherapy, later recognized as harboring a novel t(15;19) chromosomal abnormality [6]. Diagnosis relies on diffuse nuclear NUT immunostaining, typically speckled with the C52 antibody, and can be confirmed by immunohistochemistry, fluorescence in situ hybridization, reverse-transcription polymerase chain reaction, or next-generation sequencing [7]. Nuclear staining in more than 50% of tumor cells is considered highly sensitive and specific and has improved recognition of this rare tumor [1,4,8,9].

The true prevalence of NC is unknown, partly because the disease remains underdiagnosed owing to its histologic overlap with other poorly differentiated malignancies, including squamous cell carcinoma, lymphoma, neuroendocrine tumors, and sarcomas [10]. The International NUT Midline Carcinoma Registry reports approximately 20 new cases per year [1]. NC affects both males and females and may occur across a broad age range, from infancy to older adulthood, although it is more frequently reported in adolescents and young adults, with a median age of approximately 16–22 years [2,10].

Although NC classically arises from midline structures, particularly the thorax (about 51%) and head and neck region (41%), it can originate in virtually any anatomical site, including the ocular globe, salivary glands, brain, kidney, stomach, adrenal gland, pancreas, soft tissues, and bone [11]. For this reason, the term “midline” is no longer included in the current nomenclature [12].

Given its rarity and heterogeneity, no standardized effective treatment has been established. Most patients receive multimodal therapy, including multidrug chemotherapy, radiotherapy, and, when feasible, surgical resection. NC may show initial chemo- and radiosensitivity; however, responses are often transient, and relapses or rapid progression are common [4,10,13]. Prognosis remains extremely poor, with a median survival of only 5 months and 1- and 5-year OS of 25% and 7%, respectively, reflecting a highly aggressive malignancy [7], although occasional long-term survivors have been described after early diagnosis and curative-intent multimodal treatment [1].

Baseline stage appears to be a major prognostic factor, with markedly worse outcomes in patients presenting with metastatic disease compared with those with non-metastatic disease [7]. Because of its aggressive clinical course, early and accurate imaging assessment is essential to define disease extent, guide treatment planning, and document treatment-related imaging changes. However, imaging findings of NC have not been extensively described, and no specific radiologic features have been established.

Contrast-enhanced computed tomography (CT) is generally recommended for initial staging [13]. Magnetic resonance imaging (MRI) provides additional information, especially for soft-tissue definition [1]. Nevertheless, because NC often presents as a necrotic, heterogeneous, and disseminated tumor, morphological imaging alone may not fully capture viable tumor burden or occult metastatic disease.

In this setting, [18F]fluorodeoxyglucose positron emission tomography/computed tomography ([18F]FDG PET/CT) provides whole-body metabolic assessment in NC and can depict FDG-avid primary and metastatic disease [14]. Published evidence suggests potential PET applications in biopsy guidance, staging, restaging, and treatment-related metabolic assessment [1,15]. However, the literature remains sparse and heterogeneous, consisting mainly of isolated case reports and imaging-focused publications; consequently, the typical metabolic imaging presentation, the optimal timing of serial PET/CT, and the clinical relevance of PET-derived information remain undefined [16].

Here, we present three patients with NC evaluated at our institution with serial [18F]FDG PET/CT, complemented by a narrative review of the published PET imaging reports. Our aim is to describe the case-level [18F]FDG PET/CT findings observed in our cases and to discuss them in the context of the available literature on NUT carcinoma.

## 2. Case Series and Methods

We report three retrospective, descriptive cases of NC involving two pediatric patients and one young adult, each with distinct primary sites, disease extent at diagnosis, treatment strategy, and clinical outcomes. All patients underwent [18F]FDG PET/CT at clinically determined time points during their disease course at the Policlinic of Bari. Morphological imaging was also collected for the three cases when available. Case-level PET/CT observations are reported descriptively.

Written informed consent for imaging and the use of clinical data for research purposes was obtained from all patients or their legal guardians.

### 2.1. [18F]FDG PET/CT Acquisition, Analysis and Interpretation

All PET/CT examinations were performed on the Discovery IQ PET/CT system (GE Healthcare Technologies, Milwaukee, WI, USA) using the same scanner for baseline and serial evaluation. All patients fasted for at least 6 h and had blood glucose levels below 200 mg/dL before tracer administration. An intravenous injection of 2.5 MBq/kg of [18F]FDG was administered and PET/CT scanning was performed 60 min after injection. Non-contrast CT and subsequent PET acquisitions were performed in the supine position from the skull base to the mid-thigh; when indicated according to the clinical history and disease distribution, the acquisition range was extended from the vertex to the feet.

PET data were acquired in three-dimensional mode for 2.5 min per bed position. PET images were reconstructed using Q.Clear (GE Healthcare), an iterative Bayesian penalized-likelihood reconstruction algorithm based on block-sequential regularized expectation maximization (BSREM), with a β value of 350, and no additional post-reconstruction filtering was applied. Images were reconstructed using a 70 cm display field of view (DFOV), a 256 × 256 matrix, an in-plane pixel size of 2.73 mm, and a reconstructed slice thickness of 2.5 mm. CT-based attenuation correction was performed by the PET/CT system using the corresponding CT attenuation map (Q.AC WideView reconstruction), and CT images were also used for anatomical localization and image interpretation.

CT acquisition parameters included a slice collimation of 20 mm, a table feed of 18.75 mm per rotation, a pitch of 0.938:1, a gantry rotation time of 0.5 s/rot, a tube voltage of 120 kVp, and automatic tube-current modulation up to 140 mA. CT images were reconstructed with a slice thickness of 3.75 mm, a 512 × 512 matrix, a 50 cm DFOV, and the Standard WideView reconstruction filter.

Image analysis was performed on a dedicated workstation (AW Server 4.7, GE Healthcare, Milwaukee, WI, USA). All PET/CT examinations were reviewed by an experienced nuclear medicine physician (C.F.), who performed visual interpretation and semiquantitative analysis. Pathological primary, nodal and distant metastatic foci of FDG uptake were individually documented; physiologic or clearly non-neoplastic uptake was excluded from lesion detection. PET images were evaluated semiquantitatively using SUVmax.

In addition, baseline metabolic tumor volume (MTV) and total lesion glycolysis (TLG) were calculated as exploratory, descriptive imaging measures rather than validated quantitative biomarkers. For this purpose, semiquantitative volumetric analysis was carried out using PET VCAR software version 12.3 (GE Healthcare). All lesions were semiautomatically segmented using a relative threshold of 42% of lesion SUVmax. This threshold was selected as a single reproducible and pragmatic method for retrospective quantification and because relative thresholds in the 40–50% SUVmax range, including 42%, have been widely used for PET-derived MTV/TLG measurements [17,18]. Semiautomatically generated volumes of interest were visually reviewed by the same reader, with manual exclusion of physiologic uptake when necessary.

Moreover, whole-body MTV (MTVwb) was calculated as the sum of lesion-level MTV values expressed in cm^3^, and whole-body TLG (TLGwb) as the sum of lesion-level TLG values, with TLG calculated as MTV × SUVmean. No diagnostic or prognostic cut-off was prespecified, and MTV/TLG values were not used to infer prognosis, risk stratification, or treatment decisions in this three-patient series.

Serial PET/CT examinations were interpreted descriptively by comparing the distribution and intensity of FDG uptake across imaging time points. Longitudinal changes were reported as decreased or increased FDG uptake, disappearance of previously FDG-avid lesions or appearance of new FDG-avid lesions, and changes in disease distribution.

### 2.2. Narrative Literature Review

A structured narrative review was performed using PubMed/MEDLINE and Scopus to identify reports containing PET imaging information relevant to NUT carcinoma diagnosis, staging, restaging, longitudinal treatment-related PET changes, and follow-up. The search had no beginning date limit and was updated through June 2026. Free-text keywords and controlled vocabulary related to NUT carcinoma (including “NUT carcinoma”, “NUT midline carcinoma”, and “NUTM1”) were combined with PET-related terms (“PET”, “PET/CT”, “FDG”, “fluorodeoxyglucose”) and, for alternative tracers, terms including “FAPI”, “FAP”, and “DOTA”. Reviews, case series, and case reports were eligible when they described NUT carcinoma and contained PET/CT information relevant to the aim of this report. Duplicate publications and conference abstracts without extractable data were excluded; reference lists of eligible articles were also screened. Non-English full texts were excluded when a reliable translation was not available. Two authors (C.B. and A.G.N.) independently screened the retrieved records and extracted anatomical site and stage, treatment strategy, PET/CT timing and findings, and outcome, resolving discrepancies by consensus. Owing to marked heterogeneity and the predominance of case-level evidence, results were synthesized qualitatively without meta-analysis.

Twenty-three publications met the narrative scope and were included in the structured narrative review.

### 2.3. Clinical Case #1: Chest Wall NUT Carcinoma

**Figure 1:**
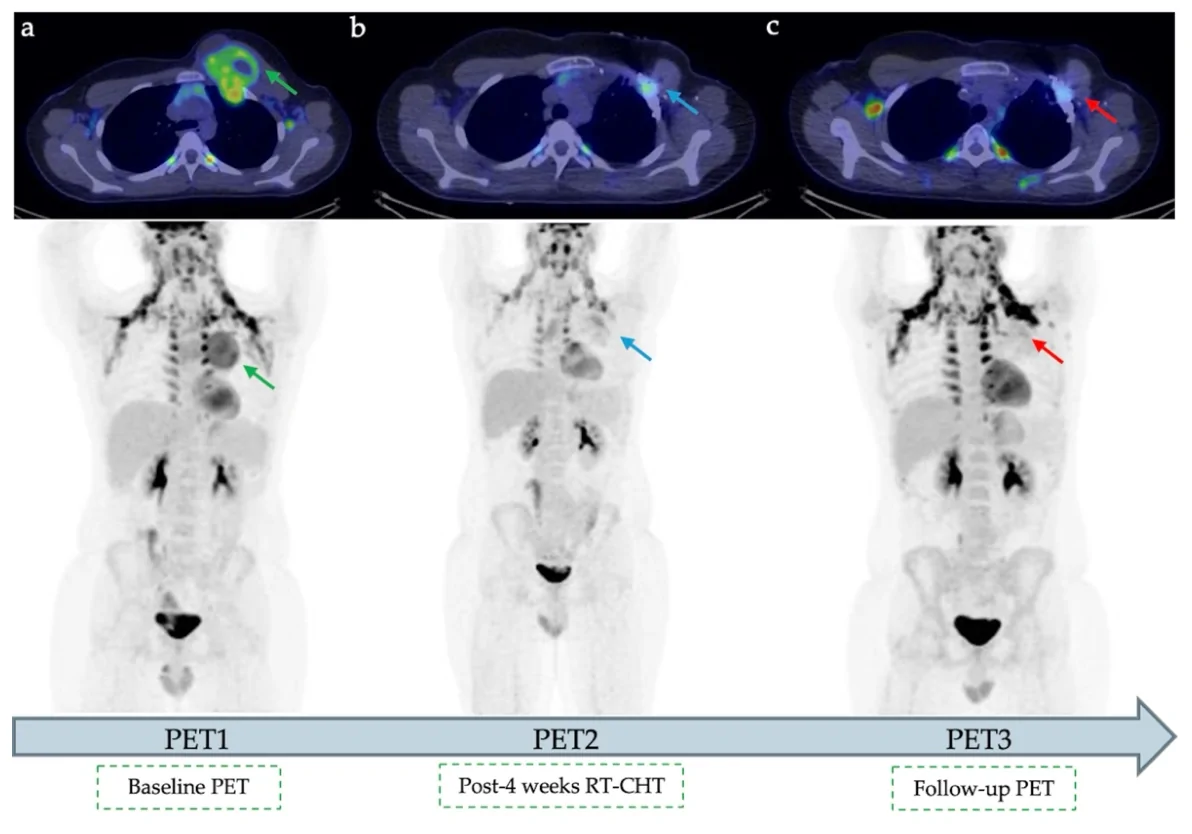
Serial [18F]FDG PET/CT in a 13-year-old male patient with NUT carcinoma of the left anterior chest wall (clinical case #1). Upper row: axial fused PET/CT images; lower row: maximum-intensity projection images (MIP). (**a**) Baseline PET/CT (PET1) showed heterogeneous FDG uptake in the primary left anterior chest wall lesion (green arrows, SUVmax 7.2, MTV 126 cm^3^, TLG 580.57 g/mL × cm^3^), located in the first left intercostal space, adjacent to the parietal pleura. Bilateral FDG uptake was observed in the retro-nuchal, supraclavicular, retroclavicular, paravertebral, and axillary regions, consistent with activation of brown adipose tissue. (**b**) Post-surgical and adjuvant therapy PET/CT (PET2) demonstrated markedly decreased and heterogeneous FDG uptake in the treated chest wall region (blue arrows, SUVmax 3.7). (**c**) Follow-up PET/CT (PET3) showed persistent inhomogeneous FDG uptake in the left anterior chest wall region adjacent to the prosthetic reconstruction and extending to the contiguous soft tissues (red arrows, SUVmax 3.2). Mild FDG uptake was observed in the anterior left rib fractures (SUVmax 2.5), without abnormal FDG-avid findings suggestive of recurrent disease.

A 13-year-old male patient presented with a painless swelling of the anterior chest wall. Chest MRI revealed an oval, heterogeneous solid soft-tissue mass (65 × 40 × 30 mm), located adjacent to the left parietal pleura, and extending anteriorly through the first intercostal space with a partially cystic component. The clinical and molecular characteristics, baseline CT imaging findings, treatment course, and clinical outcome of this patient were previously reported [19]. The present manuscript focuses on PET-specific information: the serial post-treatment and follow-up PET/CT findings and the semiquantitative and volumetric analyses are presented here for the first time. Baseline [18F]FDG PET/CT (PET1, Figure 1a) demonstrated heterogeneous FDG uptake within the primary lesion in the left anterior chest wall, together with mildly FDG-avid reactive left axillary lymph nodes.

A tru-cut biopsy established the diagnosis of NUT carcinoma, showing focal, weak nuclear NUT expression in more than 50% of neoplastic cells and a Ki-67 proliferation index of 40%. FISH analysis was negative for the canonical BRD4:NUTM1 fusion, whereas targeted RNA sequencing revealed a novel NSMCE2:NUTM1 gene fusion.

The patient subsequently underwent multimodal treatment. En bloc surgical resection of the anterior chest wall was performed, including the first and second ribs, intercostal muscles, pectoral muscles, overlying skin, and subcutaneous tissue surrounding the lesion, together with left axillary lymph node dissection. Chest wall reconstruction was achieved using a titanium plate to restore the resected bony structures. Histological examination confirmed complete tumor resection with negative surgical margins (R0) and no evidence of lymph node involvement.

Postoperatively, adjuvant radiotherapy (RT) was delivered to the tumor bed using volumetric modulated arc therapy (VMAT) with a total dose of 61.2 Gy in 35 fractions. Concurrent chemotherapy (CHT) consisted of alternating VAI (vincristine, doxorubicin, and ifosfamide) and PAI (cisplatin, doxorubicin, and ifosfamide) regimens, administered every 21 days (11 cycles).

Following completion of chemoradiotherapy, [18F]FDG PET/CT (PET2, Figure 1b) demonstrated a marked reduction in FDG uptake within the treated chest wall lesion. The residual low-grade metabolic activity was considered consistent with post-treatment inflammatory changes. During follow-up, contrast-enhanced CT and [18F]FDG PET/CT examinations confirmed only treatment-related changes, including pulmonary fibrosis within the irradiated field and a pathological fracture of the anterior arch of the left third rib, without imaging evidence of metabolically active residual or recurrent disease.

At the last available follow-up, 30 months from diagnosis, the patient remained alive and in complete clinical remission.

### 2.4. Clinical Case #2: Head and Neck NUT Carcinoma

A 20-year-old female patient presented with a rapidly enlarging and painful left submandibular mass, initially raising suspicion of a lymphoproliferative disorder. Neck MRI revealed a large solid left submandibular lesion measuring 76 × 76 × 67 mm, characterized by heterogeneous signal intensity and internal necrotic areas. The mass was closely related to the left submandibular gland, mylohyoid muscle, and adjacent neurovascular structures, resulting in displacement of the tongue and pharyngolaryngeal compartments toward the right side, with associated narrowing of the upper airway. Partially confluent left level I–III cervical lymphadenopathies were also observed.

**Figure 2:**
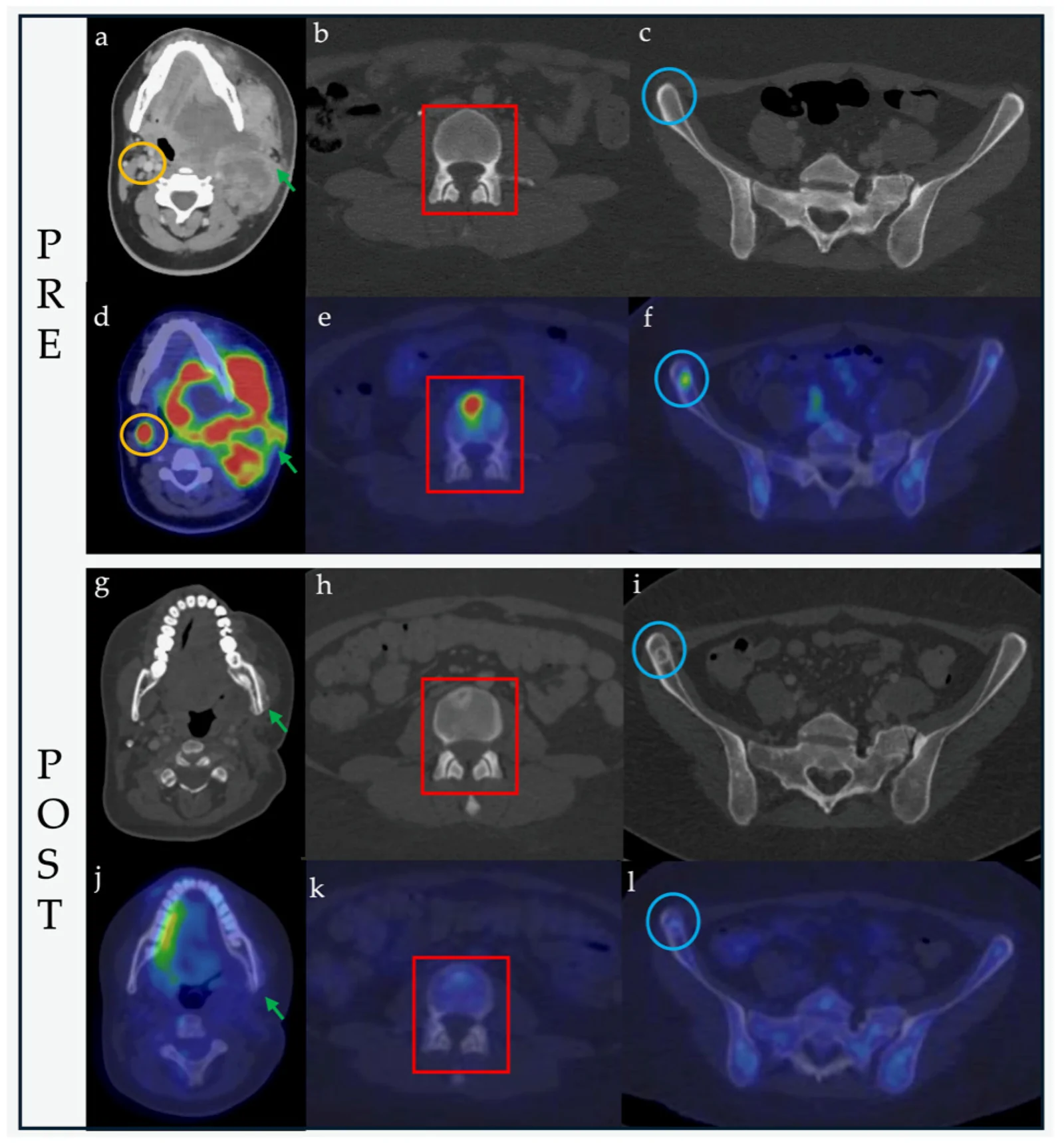
Baseline and post-chemotherapy imaging findings in a 20-year-old female patient with head and neck NUT carcinoma. Baseline axial contrast-enhanced CT images (**a**–**c**) and corresponding fused PET/CT images (**d**–**f**) show a large left submandibular mass measuring 79 × 104 × 98 mm ((**a**,**d**) green arrows, SUVmax 29.1), characterized by extensive internal necrosis. The lesion infiltrates the left carotid–jugular neurovascular bundle, with neoplastic thrombosis of the left internal jugular vein, and extends cranially to the oro- and nasopharynx, resulting in upper-airway stenosis and contralateral displacement. Necrotic left level III–IV cervical lymphadenopathies measuring up to 3 cm are visible on CT, while PET/CT demonstrates bilateral FDG-avid cervical nodal involvement ((**a**,**d**) orange circles; SUVmax 22.9). Multiple skeletal FDG-avid metastases are detected despite the absence of abnormalities on CT, predominantly involving L3 ((**b**,**e**); red boxes, SUVmax 14.5), and the right anterior superior iliac spine lesion ((**c**,**f**); blue circles, SUVmax 5.8). After four cycles of chemotherapy, axial CT images (**g**–**i**) and corresponding fused PET/CT images (**j**–**l**) demonstrate a marked reduction in the size and FDG uptake of the primary lesion ((**g**,**j**) green arrows, SUVmax 2.2), with residual small ipsilateral cervical lymphadenopathies and a periosteal reaction of the left mandible visible on CT (**g**, green arrow). PET/CT shows no residual FDG uptake at the previously involved nodal and skeletal sites (**k**, red boxe; **l**, blue circle) whereas CT reveals bone remodeling at the previously involved skeletal sites, characterized by an osteolytic core and a peripheral sclerotic rim (**h**, red boxe; **i**, blue circle).

Subsequent contrast-enhanced total-body CT (Figure 2a–c) confirmed the presence of an enlarged left submandibular mass with extensive central necrosis, associated with necrotic lymphadenopathy at left cervical levels III–IV. A pulmonary pseudonodular opacity measuring 8 mm, with a small ground-glass halo in the right upper lobe, was considered of uncertain significance.

**Figure 3:**
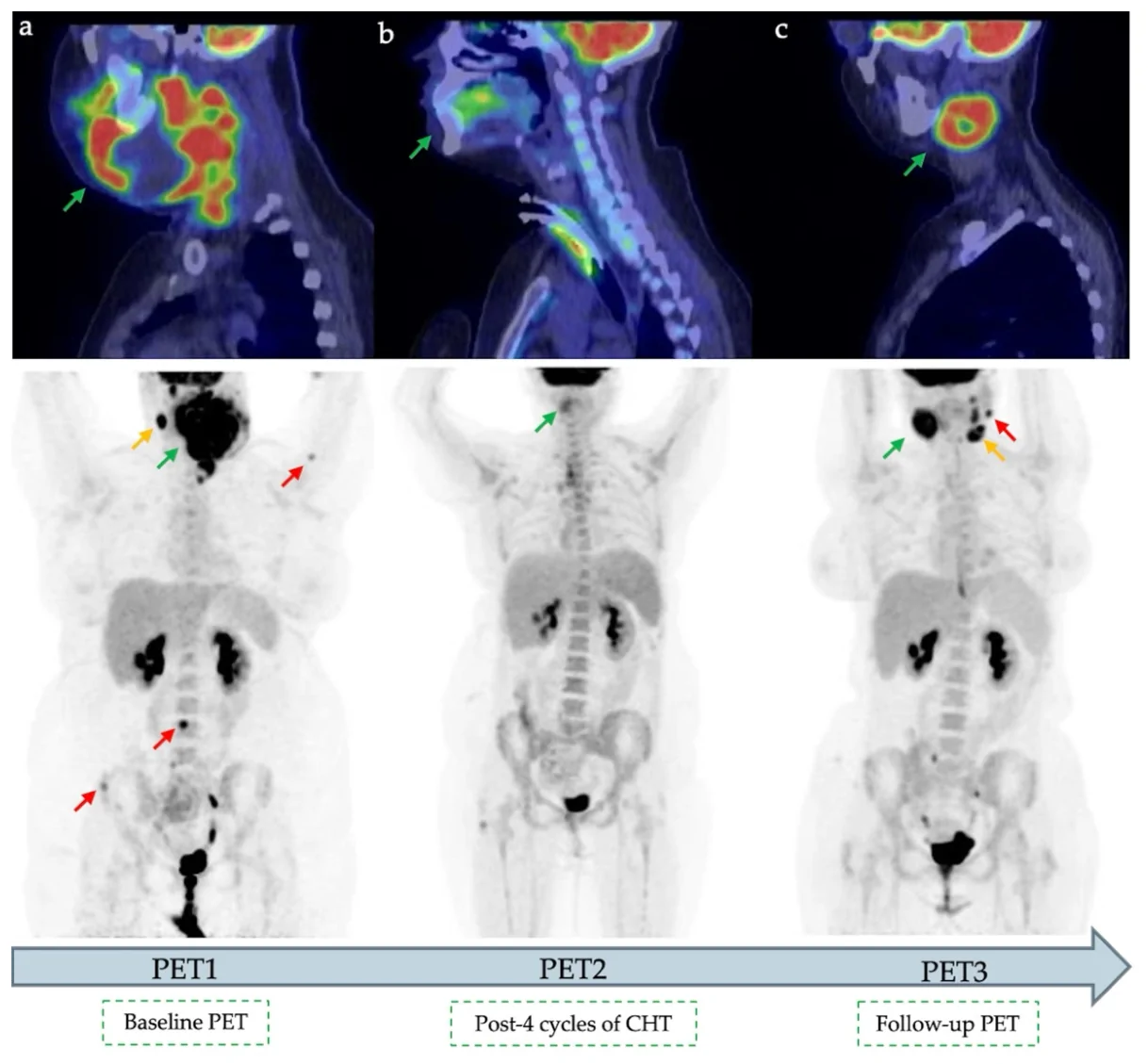
Serial [18F]FDG PET/CT in a 20-year-old female patient with NUT carcinoma of the head and neck region (clinical case #2). Upper row: sagittal fused PET/CT images; lower row: maximum-intensity projection images (MIP). (**a**) Baseline PET/CT showed intense FDG uptake in the left submandibular primary lesion (green arrows, SUVmax 29.1, MTV 416 cm^3^, TLG 3987.90 g/mL × cm^3^), bilateral cervical nodal disease (orange arrow, right side SUVmax 22.9), and multiple FDG-avid skeletal metastases (red arrows), involving the left humerus (SUVmax 6.9), D12 (SUVmax 4.2), L3 (SUVmax 14.5), and the right anterior superior iliac spine lesion (SUVmax 5.8); whole-body MTV (MTVwb) 424.364 cm^3^, whole-body TLG (TLGwb) 4045.87 g/mL × cm^3^. (**b**) PET/CT after chemotherapy showed a marked reduction in the metabolic activity of the primary lesion (green arrow, SUVmax 2.2), with no residual FDG uptake at the previously involved nodal and skeletal sites. (**c**) Follow-up PET/CT showed increased FDG uptake in the locoregional disease (green arrows, SUVmax 14.0), cervical lymph nodes (orange arrow, left side SUVmax 13.9), and left mandible (red arrow, SUVmax 9.6).

Baseline [18F]FDG PET/CT (PET1, Figure 2d–f and Figure 3a) demonstrated intense and heterogeneous FDG uptake within the left submandibular primary lesion, characterized by extensive central necrosis, as well as increased uptake in bilateral cervical lymphadenopathies. In addition, PET/CT identified multiple areas of increased skeletal FDG uptake consistent with metastatic involvement, despite the absence of corresponding morphological abnormalities on the CT component.

Biopsy of the left laterocervical mass confirmed a poorly differentiated NC with focal squamous differentiation and a high proliferative index (Ki-67 > 80%). The diagnosis was supported by NUT immunohistochemistry; the exact percentage of NUT-positive tumor nuclei was not available in the retrospective record. Additional molecular characterization of the NUTM1 rearrangement and fusion partner was not available for this patient.

Given the risk of upper airway obstruction, the patient underwent tracheostomy and subsequently received four cycles of systemic chemotherapy with docetaxel, cisplatin, and 5-fluorouracil. After four cycles of chemotherapy, [18F]FDG PET/CT (PET2, Figure 2j–l and Figure 3b) showed a marked decrease in both the size and metabolic activity of the primary tumor, with no residual FDG uptake at the previously involved nodal and skeletal sites. CT confirmed a substantial reduction in primary tumor volume but demonstrated multiple skeletal lesions characterized by an osteolytic center with a peripheral sclerotic rim. These findings were considered equivocal, potentially reflecting either treatment-related bone remodeling changes or residual metastatic disease (Figure 2g–i).

Two months later, the patient experienced clinical deterioration with progressive respiratory distress and Klebsiella pneumoniae bacteremia, prompting further disease assessment. CT showed new right pulmonary nodules suspicious for metastases and cervical nodal involvement, including the appearance of a large necrotic right cervical lymph node. Biopsy of the latter lesion confirmed metastasis of NUT carcinoma. [18F]FDG PET/CT (PET3, Figure 3c) demonstrated increased FDG uptake at the primary tumor site and cervical lymph nodes, together with FDG-avid involvement of the left mandible.

The patient subsequently underwent combined chemoradiotherapy followed by capecitabine maintenance treatment. Despite further therapy, she developed paraplegia and progressive muscle weakness secondary to widespread pulmonary and skeletal disease progression, with CT documenting extension into the spinal canal at the D10–D11 level. Given her poor performance status, neurosurgical intervention was not feasible, and single-fraction palliative radiotherapy was administered. Shortly thereafter, the patient developed septic shock and died 11 months after diagnosis.

### 2.5. Clinical Case #3: Mediastinal NUT Carcinoma

**Figure 4:**
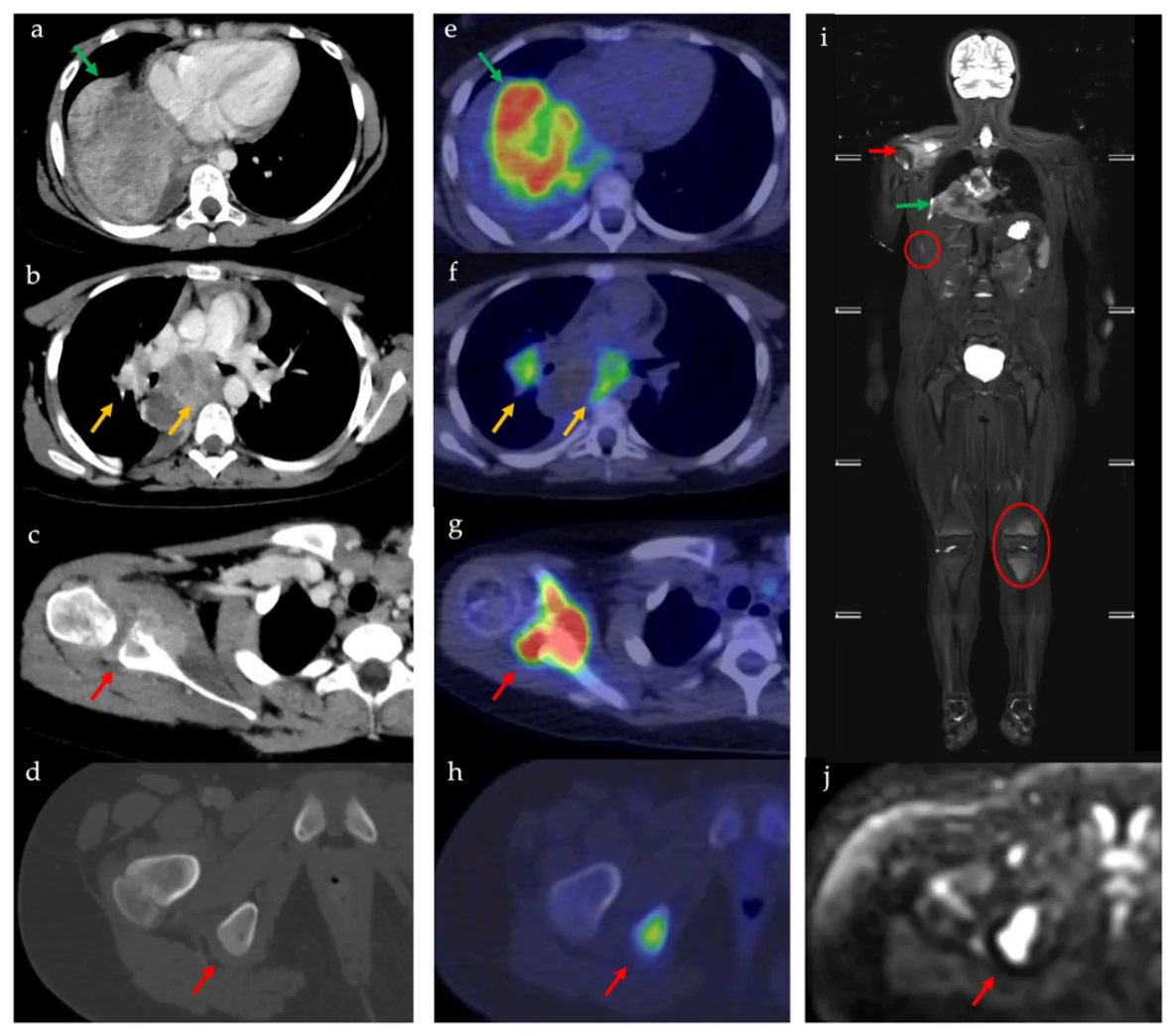
Baseline multimodality imaging in an 8-year-old male patient with mediastinal NUT carcinoma. Contrast-enhanced CT images (**a**–**d**) show a large heterogeneous right thoracic mass involving most of the middle and lower pulmonary lobes ((**a**), green arrow), with mediastinal and pericardial extension, associated with necrotic right hilar and subcarinal lymphadenopathies ((**b**), orange arrows), causing posterior compression of the adjacent tracheobronchial airway, and a destructive osteolytic lesion of the right scapula with periosteal reaction ((**c**), red arrow). No other skeletal abnormalities were visible on CT images ((**d**), red arrow). Corresponding fused [18F]FDG PET/CT images (**e**–**h**) demonstrate intense FDG uptake in the right thoracic mass ((**e**), green arrow, SUVmax 12.4), ipsilateral hilar and subcarinal lymph nodes ((**f**), orange arrows, SUVmax 12.2 and 10.4, respectively), right scapular lesion ((**g**), red arrow, SUVmax 16.3), and right ischiopubic ramus ((**h**), red arrow, SUVmax 9.5). Whole-body MRI images (**i**,**j**) demonstrate the right thoracic mass (**i**, green arrow), associated with multifocal skeletal and soft-tissue involvement, including lesions of the right scapula (**i**, red arrow),right ninth rib (**i**, red circle), right ischiopubic ramus (**j**, red arrow), distal left femur and proximal left tibia (**i**, red ellipse).

An 8-year-old male patient presented with right shoulder pain, which was investigated with a dedicated MRI of the shoulder. The examination revealed a large mass of the right scapula measuring 46 × 46 × 38 mm, with involvement of the glenoid cavity and coracoid process. The lesion was associated with displacement of the adjacent musculature and imaging features suggestive of soft-tissue infiltration. Total-body CT (Figure 4a–d) confirmed a destructive osteolytic lesion of the right scapula and identified a large heterogeneous mass in the right mediastinum, involving the middle and lower pulmonary lobes. Enlarged necrotic ipsilateral hilar and subcarinal lymph nodes were also observed, with associated compression of the adjacent tracheobronchial structures. Biopsy of the scapular lesion demonstrated metastatic involvement from a NUT carcinoma arising from a mediastinal/pulmonary primary site. The diagnosis was supported by NUT immunohistochemistry; detailed quantitative immunohistochemical results were not available in the retrospective record, and additional molecular characterization of the NUTM1 rearrangement or fusion partner was unavailable for this patient.

**Figure 5:**
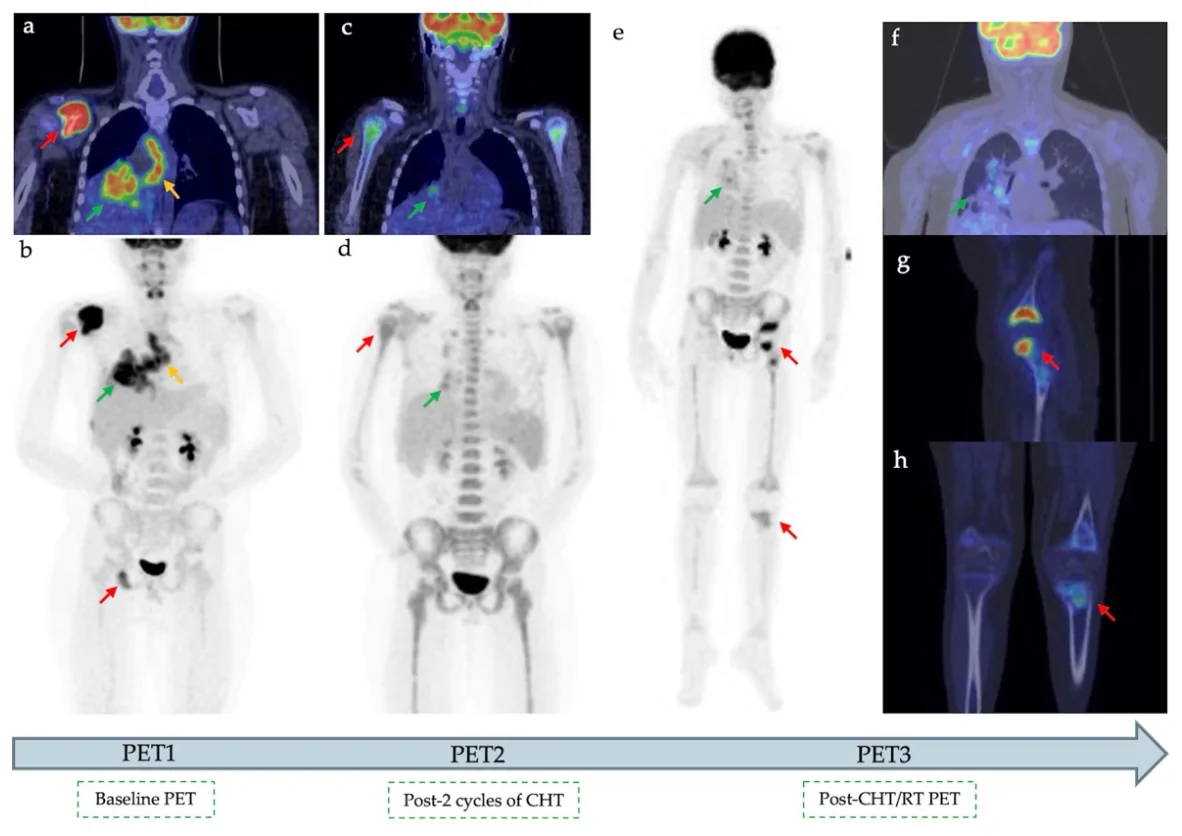
Serial [18F]FDG PET/CT in an 8-year-old male patient with NUT carcinoma of the mediastinal region (clinical case #3). (**a**,**c**,**f**,**h**): coronal; (**g**): sagittal fused PET/CT images; (**b**,**d**,**e**): maximum-intensity projection images (MIP). (**a**,**b**) Baseline PET/CT showed FDG-avid mediastinal/right pulmonary disease (green arrows, SUVmax 12.4, MTV 114 cm^3^, TLG 832.74 g/mL × cm^3^), nodal disease (orange arrows, right hilar node SUVmax 12.2), and skeletal metastases (red arrows, right ischiopubic ramus and scapula SUVmax 9.5–16.3); whole-body MTV (MTVwb) 152.17 cm^3^, whole-body TLG (TLGwb) 1224.6 g/mL × cm^3^. (**c**,**d**) Interim PET/CT after 2 cycles of chemotherapy showed markedly lower FDG uptake in the right scapula (red arrows, SUVmax 4.0) and mediastinal mass (green arrows, SUVmax 4.8). (**e**–**h**) Post-treatment PET/CT showed persistently reduced FDG uptake in pulmonary and nodal disease (green arrows), together with multiple FDG-avid skeletal metastases, including the left humeral head, left iliac wing, left acetabulum, left femoral head and intertrochanteric region, and left tibial region (red arrows, SUVmax range 3.7–15.8).

Baseline [18F]FDG PET/CT (PET1, Figure 4e–h and Figure 5a,b) showed intense FDG uptake within the primary thoracic mass, mediastinal lymphadenopathies, and the scapular metastatic lesion. An additional metabolically active skeletal lesion was detected in the right ischiopubic ramus. Whole-body MRI (Figure 4i,j) was subsequently performed to further characterize this PET skeletal finding, confirming multifocal skeletal and soft-tissue involvement, including additional lesions.

Systemic chemotherapy with VDC (vincristine, doxorubicin, and cyclophosphamide) alternating with IE (ifosfamide and etoposide) was initiated.

After two cycles, restaging CT and MRI showed a reduction in the size of the primary pulmonary mass, mediastinal lymphadenopathies, and scapular lesion. Interim [18F]FDG PET/CT showed a marked reduction in FDG uptake at the main involved disease sites (Figure 5c,d).

The patient continued systemic therapy and received radiotherapy to the right pulmonary primary lesion and scapular metastasis, followed by irradiation of additional skeletal lesions involving the right ninth rib, left femur, and tibia (total dose delivered over 26 fractions).

One month after completion of chemoradiotherapy, chest CT showed further size reduction in the right pulmonary mass and mediastinal lymphadenopathies. Conversely, MRI demonstrated new bone lesions involving the left femur, left iliac wing, acetabulum, gluteus minimus muscle, and right sacral ala. At post-treatment assessment, [18F]FDG PET/CT (PET3, Figure 5e–h) showed persistently reduced FDG uptake in pulmonary and nodal disease, while multiple FDG-avid skeletal lesions were observed.

Following disease progression, the patient died 12 months after the initial diagnosis.

The main clinical characteristics, imaging timing points, baseline PET/CT semiquantitative analysis, serial [18F]FDG PET/CT findings, outcomes, and case-level PET/CT observations of the three cases are summarized in Table 1.

## 3. Discussion and Narrative Literature Review

The available literature on [18F]FDG PET/CT in NC remains very limited and consists predominantly of isolated case reports and small retrospective series. Consequently, the metabolic imaging characteristics of this rare tumor and the optimal timing of PET/CT during the disease course remain poorly defined. In this context, the present three cases are used to describe observed PET/CT findings and serial metabolic changes in NC and to discuss reported PET applications in staging, restaging, biopsy planning, longitudinal disease assessment, and follow-up. The clinical characteristics, PET imaging findings, treatment context, and outcomes of NC patients identified through the structured narrative review are summarized in Table 2 (the Virarkar et al. publication is displayed in two site-specific rows according to the primary site).

Across the published literature, [18F]FDG PET/CT has been used alongside CT and MRI to characterize NUT carcinoma and its locoregional and distant extent [20]. Contrast-enhanced CT remains the mainstay of anatomical staging, typically showing a hypoattenuating, heterogeneously enhancing infiltrative mass with poorly defined margins, internal necrosis, and local invasion. MRI provides superior soft-tissue characterization, particularly in head and neck tumors and when skull-base extension, bone marrow infiltration, or cardiac, vascular, perineural, and spinal canal involvement is suspected [10,14]. These morphologic features may overlap with those of other high-grade malignancies, including lymphoma, sarcoma, and poorly differentiated carcinoma [21]. Definitive diagnosis relies on NUT immunohistochemistry, with molecular testing for a NUTM1 rearrangement used for confirmation or fusion-partner characterization when appropriate. In this setting, PET/CT should be regarded as a complementary imaging modality that provides whole-body metabolic information within multidisciplinary disease assessment [9,10,21,22].

Tumor necrosis is a frequent feature of NUT carcinoma, particularly in large and aggressive lesions, and represents a relevant challenge for imaging interpretation. Necrotic tumors may demonstrate heterogeneous FDG distribution, with intense uptake in viable peripheral components surrounding a relatively photopenic necrotic core [16,23]. Recognition of this pattern may help identify metabolically active tumor regions and may support biopsy targeting in selected cases [5].

Skeletal involvement represents another important imaging feature of NC. In our second patient, baseline PET/CT identified multiple FDG-avid skeletal lesions without corresponding structural abnormalities on CT. After therapy, the disappearance of abnormal FDG uptake at these sites helped contextualize subsequently visible bone remodeling on CT as a possible post-treatment change. In our third patient, PET/CT identified an additional ischiopubic skeletal lesion that prompted whole-body MRI, which confirmed multifocal skeletal involvement. Similarly, previous reports have described FDG-avid lytic, non-osteoblastic, or clinically unexpected skeletal lesions that were occult or less detectable on other imaging modalities [12,13,21,24]. Although our three cases and the available case-level literature do not allow estimation of sensitivity, specificity, incremental detection rate, or comparison against a reference standard, these findings illustrate the complementary role of [18F]FDG PET/CT within a multimodality skeletal imaging approach alongside CT and MRI.

Our cases are consistent with previous reports showing that NC lesions are frequently FDG-avid. However, the reported intensity of FDG uptake is highly variable, and no validated SUVmax threshold has been established for the diagnosis of NC or for its differentiation from other malignant or inflammatory conditions. Chang et al. described 10 patients with pulmonary NC, and all evaluable primary tumors, nodal lesions, and distant metastases were FDG-avid, with SUVmax ranging from 5 to 40 (mean SUVmax, 12) [25]. In a broader literature review, Huang et al. reported an SUVmax range of 5–40 (mean, 12.8) for pulmonary NC and 4.5–64.1 (mean, 13.8) for extrapulmonary disease [9]. Similarly, our three patients also showed substantial variability in metabolic activity across both primary and secondary lesions, with SUVmax values ranging from 7.2 in the anterior chest wall lesion to 29.1 in the head and neck primary tumor and from 4.2 to 22.9 for metastatic sites (lymph nodes and bone).

The observed variability in FDG uptake, together with the heterogeneous and frequently necrotic appearance of NUT carcinoma, highlights the need to interpret SUVmax in the broader context of lesion morphology, disease distribution, treatment strategy, and imaging timing. As SUVmax reflects only the voxel with the highest FDG uptake, it does not account for the overall extent of metabolically active disease. This provided the rationale for exploring volumetric PET parameters in our series. In other FDG-avid malignancies, particularly lymphomas, MTV and TLG have been extensively investigated as complementary quantitative metrics of metabolically active tumor burden, with MTV reflecting the volume of FDG-avid disease and TLG integrating this volume with mean FDG uptake [26]. Accordingly, we explored MTV and TLG descriptively to provide an additional quantitative characterization of baseline metabolic disease in NUT carcinoma. However, these parameters are method-dependent and have not yet been validated as biomarkers in NUT carcinoma; therefore, in the present series they were used exclusively for descriptive characterization of the three baseline examinations.

In our three cases, primary-tumor MTV ranged from 114 to 416 cm^3^, and primary-tumor TLG from 580.57 to 3987.90 g/mL × cm^3^. In the two patients with metastatic disease, MTVwb ranged from 152.17 to 424.364 cm^3^, and TLGwb from 1224.6 to 4045.87 g/mL × cm^3^. These values provide descriptive quantitative data for future investigations of volumetric and glycolytic PET parameters in larger cohorts.

To date, baseline disease extent is among the most consistently reported prognostic factors in NC, with substantially poorer outcomes in patients with metastatic disease than in those with non-metastatic disease [7]. In our series, Case 1 had localized disease and remained alive in complete clinical remission at 30 months, whereas Cases 2 and 3 had metastatic disease and died 11 and 12 months after diagnosis, respectively. These case-level observations are descriptive and do not permit prognostic inference.

The published literature further illustrates the complexity of prognostic interpretation in NC and underscores the dominant role of clinical context. Selected patients with localized or locoregionally controlled disease have achieved sustained disease control after curative-intent multimodal therapy [27,28], whereas Crocetta et al. described rapid recurrence and dissemination despite an initially non-metastatic PET/CT [29]. These observations indicate that neither SUVmax nor the absence of metastatic disease on a single examination should be regarded as a stand-alone prognostic marker.

Although MTV and TLG were used in the present series solely as feasibility-oriented, descriptive measurements, these parameters could be investigated in larger cohorts as quantitative estimates of metabolically active disease burden alongside established clinical prognostic factors.

A further clinically relevant question is the lack of standard imaging time points for PET/CT evaluation in this rare disease. The serial examinations in our cases were performed at heterogeneous, clinically driven time points: after different numbers of chemotherapy cycles, at different intervals after therapy, and in various treatment contexts. They should therefore be regarded as illustrative longitudinal case observations. In Case 3, an early reduction in FDG uptake was followed by rapid, site-specific progression; similar discordant or transient metabolic patterns have been described in individual reports by Rosenbaum et al., Niederkohr et al., and Ciftci et al. [5,13,14]. These observations show that serial PET/CT can document changing and heterogeneous metabolic disease patterns in selected patients, but they cannot establish the optimal timing of imaging, predict treatment response, or demonstrate an association between early changes in FDG uptake and survival. Prospective studies using predefined imaging time points and standardized longitudinal PET interpretation methods would be required to address these questions. Serial whole-body [18F]FDG PET/CT has been used to document the appearance of uncommon metastatic sites in individual reports, including the case by Gupta et al. that described the evolution of thoracic disease to muscular, pulmonary, and cerebral involvement [30].

NC is associated with a poor prognosis because of its rapid progression, frequent metastatic presentation, high recurrence rate, and limited response to available treatments. Chang et al. reported more favorable outcomes with no evidence of recurrence over follow-up periods of 6–35 months in patients with lower-stage pulmonary disease, whereas those with advanced disease died within 3–12 months [25]. Similarly, Xie et al. observed a median overall survival of 4.1 months (range, 1.5–26.7 months) in patients with advanced pulmonary or tracheal NC, although selected patients receiving radiotherapy and immune checkpoint inhibitors survived beyond one year [31]. A patient data analysis of 119 cases reported 1-, 2-, and 5-year overall survival rates of 31.7%, 8.7%, and 8.7%, respectively, in patients with non-metastatic disease, compared with 9.8%, 0%, and 0% in those with metastatic disease at diagnosis (*p* = 0.004) [32]. In our series, the patient with localized disease remained alive and in complete clinical remission at 30 months from diagnosis, whereas the two patients with metastatic disease died 11 and 12 months after diagnosis, respectively. These case-level outcomes resemble stage-related patterns reported in the literature but do not provide independent evidence of a prognostic association.

Although the present study focuses on [18F]FDG PET/CT, a limited number of recent reports have investigated alternative PET radiotracers in NC. FAP-targeted tracers, including [68Ga]Ga-FAPI-04 and [68Ga]Ga-FAP-2286, have shown high tumor uptake and favorable tumor-to-background contrast in individual NC presentations, with possible value for lesion depiction; in one mediastinal case, [68Ga]Ga-FAPI-04 delineated pleural involvement more clearly than [18F]FDG PET/CT [8,33,34]. [68Ga]Ga-DOTA-IBA has also demonstrated uptake in skeletal and extraosseous lesions, raising interest in a possible theragnostic application with [177Lu]Lu-DOTA-IBA, although therapeutic efficacy remains unproven [35]. Evidence for non-FDG tracers remains limited to isolated case reports and is insufficient to support their routine use or comparative performance claims. At present, [18F]FDG remains the most extensively reported PET radiotracer in the NC literature.

### Limitations

First, this is a retrospective descriptive series of only three patients, with major heterogeneity in age, primary site, baseline stage, resectability, treatment strategy, and outcome. Given the sample size and marked clinical heterogeneity, this report does not attempt to establish diagnostic accuracy, prognostic associations, risk stratification, treatment-selection utility, or a standardized role or optimal timing for PET/CT.

Second, serial PET/CT examinations were obtained at heterogeneous, clinically driven time points. Longitudinal PET/CT findings were described in terms of changes in FDG uptake and disease distribution, without assigning formal categorical PET response.

Third, quantitative PET measurements are method-dependent. MTV and TLG were derived using a fixed 42% SUVmax threshold that is not validated for NC [17,18]. Measurements were performed by a single reader without independent verification or interobserver analysis.

Finally, the accompanying literature synthesis is a structured narrative review. The evidence base is dominated by case reports and small series. Accordingly, all conclusions from the present manuscript should be considered illustrative and hypothesis-generating.

**Table 2:** Structured narrative summary of published reports on PET imaging in NUT carcinoma. Serial PET findings are reported descriptively, based on the imaging changes described in the source articles.

Author, Year	Age/Sex	Primary Site	PET Timing and Serial Findings	Dominant Baseline Lesion	Baseline Disease Extent	Outcome	PET-Related Observation
Niederkohr et al., 2011 [14]	13 y, F	Pulmonary NC	- Post-CHT [18F]FDG PET/CT; serial PET/CT showed initial FDG uptake decrease followed by worsening FDG-avid disease	Right lung lesion (SUVmax NR)	- Primary: right lung - Multiple bone metastases	NR	Serial PET/CT documented treatment-related metabolic changes during FDG-avid disease course
Rosenbaum et al., 2012 [13]	17 y, M	Mediastinal NC	- Interim [18F]FDG PET/CT: decreased FDG uptake in the mediastinal mass, with widespread lytic skeletal disease; - Post-CHT PET/CT: worsening FDG-avid disease	Mediastinal mass (SUVmax 4.6)	- Primary: bulky mediastinal mass (SUVmax 4.6). - Metastases: bone metastases	Died, OS 5 months	PET/CT may reveal skeletal metastases that could be underestimated by bone scintigraphy
Ciftci et al., 2015 [5]	7 y, M	Mediastinal NC	- Baseline [18F]FDG PET/CT. - Post-CHT (4 cycles) PET/CT: worsening FDG-avid disease	Right hemithorax mass (SUVmax 5.8)	- Primary: right hemithorax mass (SUVmax 5.8). - Metastases: left cervical, mediastinal, abdominopelvic lymphadenopathies (SUVmax 2.7–5.4); liver (SUVmax 3.7) and bone metastases (SUVmax 6.6)	NR	PET/CT was used for staging, biopsy-site localization, and restaging. Serial imaging documented treatment-related changes and later worsening FDG-avid disease. In this case, moderate FDG avidity at diagnosis was followed by higher FDG uptake during disease progression
Shaikh et al., 2015 [10]	29 y, F	Head and neck NC	- Staging/post-surgical [18F]FDG PET/CT. - Restaging PET/CT: persistent residual disease and new nodal and bone metastases	Left medial canthus/left nasal cavity lesion (SUVmax NR)	- Primary: left medial canthus/nasal cavity soft tissue mass. - Metastases: bilateral cervical lymph nodes	NR	CT, MRI, and PET/CT were used for staging and restaging; serial PET/CT depicted residual disease and subsequent nodal and skeletal involvement
Perkins et al., 2017 [21]	2 y, F	Mediastinal NC	- 2 weeks after surgery [18F]FDG PET/CT - 9 weeks after surgery [18F]FDG PET/CT: worsening FDG-avid disease	Mesenteric retroperitoneal tumor (SUVmax 4.5)	- Primary: pericardial soft tissue mass (SUVmax 2.5). - Metastases: mesenteric soft tissue tumor (SUVmax 4.5)	Died, OS 11 months	[18F]FDG PET/CT was used for staging, disease-extent assessment, and longitudinal documentation of treatment-related metabolic changes
Virarkar et al., 2020 [12]	a. 43 y, F b. 48 y, F c. 28 y, F d. 52 y, M	Pulmonary NC	- Baseline [18F]FDG PET/CT	Lung mass (SUVmax NR)	a. Primary: left upper lobe lung. b. Primary: left thorax. Metastases: pleural effusion and thickening. c. Primary: left lower lobe lung. Metastases: mediastinal lymphadenopathy, left scapula. d. Primary: left lower lobe lung. Metastases: pleural effusion and thickening	NR	[18F]FDG PET/CT as part of multimodality disease-extent evaluation in pulmonary NC, with FDG-avid masses and frequent nodal/pleural findings
Virarkar et al., 2020 [12] *	a. 55 y, F b. 24 y, M c. 37 y, F d. 37 y, F e. 51 y, F f. 42 y, F	Head and neck NC	Baseline [18F]FDG PET/CT	Head and neck (SUVmax NR)	a, b, e, f. Primary: ethmoid region mass. c, d. Primary: right maxillary sinus	NR	[18F]FDG PET/CT as part of multimodality disease-extent evaluation in head and neck NC, alongside CT/MRI characterization of locally destructive and necrotic tumors for disease-extent evaluation. Head and neck NC presents as an expansile and necrotic mass, with locoregional destruction and less common necrotic lymph node involvement
Surro et al., 2020 [23]	23 y, F	Mediastinal NC	Baseline [18F]FDG PET/CT	Mediastinal mass (SUVmax 9.5)	- Primary: mediastinal/right hilar mass (SUVmax 9.5). - Metastases: right supraclavicular lymph nodes	Died, OS 20 days (pulmonary emboli as the cause of death)	[18F]FDG PET/CT delineated metabolically active disease and helped identify viable tumor around necrotic components for biopsy planning
Xie et al., 2020 [31]	7 patients; 23–74 y; 4 M/3 F	Pulmonary or tracheal NC	Baseline [18F]FDG PET/CT available in 3/7	Lung mass (SUVmax 10.6–18.6)	- Primary: pulmonary/tracheal mass. - Metastases: hilar, mediastinal and supraclavicular lymph nodes; bone and adrenal gland involvement	Median OS 4.1 months (range, 1.5–26.7); one patient alive at ≥12 months	PET documented high but variable FDG avidity and contributed to whole-body staging
Chang et al., 2021 [25]	10 patients; 18–49 y; 7 M/3 F	Pulmonary NC	- Baseline [18F]FDG PET/CT in 9/10	Pulmonary mass (SUVmax 5–40, mean 12)	- Primary: pulmonary mass - Metastases: mediastinal/hilar/paratracheal/subcarinal nodal disease in 8/10 pts, pleural seeding/effusion in 2/10, liver, adrenal gland and bone metastases in 2/10	Five lower-stage patients were in complete remission at 6–35 months; four advanced-stage patients died at 3–12 months; one patient was under treatment	A wide SUV range was reported in pulmonary NC; outcomes in this series were described according to baseline stage, with more favorable outcomes in lower-stage patients
Leeman et al., 2021 [27]	15 y, F	Head and neck NC	- Baseline [18F]FDG PET/CT; - PET/CT after 2 CHT cycles: decreased FDG uptake; - 1 month post-RT: low residual FDG (SUVmax 2.5)	Right sinonasal mass (SUVmax 8)	- Primary: right sinonasal mass with intracranial extension (T4b); - Metastases: none (N0M0)	Alive, OS 34 months after diagnosis	PET/CT documented baseline T4bN0M0 disease, decreased FDG uptake after chemotherapy, and low residual FDG uptake after radiotherapy
Crocetta et al., 2021 [29]	56 y, F	Head and neck NC	- Baseline [18F]FDG PET/CT before surgery	Right nasal fossa mass (SUVmax 15.4)	- Primary: right nasal fossa lesion. - Metastases: none	Died, OS 7 months	Intense primary FDG uptake and no distant metastases were reported at baseline, followed by early dissemination; the observation does not support prognostic inference from SUVmax
Cooper et al., 2022 [20]	7 y, M	Mediastinal NC	- Baseline [18F]FDG PET/CT	Left paratracheal lymph node (SUVmax 10.9)	- Primary: middle mediastinal mass (SUVmax 10.7) - Metastases: bilateral paratracheal lymph nodes (10.9)	NR	[18F]FDG PET/CT showed FDG-avid primary and nodal disease; no distant metastases were reported
Huang et al., 2022 [22]	58 y, F	Head and neck NC	[18F]FDG PET/CT performed 1.5 months after surgery: intense FDG uptake in recurrent disease	Septal sinus, pterygoid sinus, right nasal cavity, anterior skull-base soft tissue disease (SUVmax 64.1)	Primary: septa and pterygoid sinus, right nasal cavity, anterior skull-base, with bilateral compression of the internal rectus muscle and optic nerve, and localized bone destruction in the septal sinus and pterygoid sinus	Died, OS 17 months	CT, MRI, and PET/CT documented early postoperative recurrence; PET showed intense FDG uptake in recurrent disease
Gupta et al., 2022 [30]	49 y, M	Pulmonary NC	Baseline, restaging (after 6 and 13 months from diagnosis) [18F]FDG PET/CT	Right upper-lobe mass (SUVmax NR)	- Primary: right upper lobe mass. - Metastases: paratracheal and suprahilar lymph nodes	Died, OS 18 months	PET/CT was performed at baseline and for restaging to document disease extent and longitudinal metabolic change
Huang et al., 2023 [9]	19 y, M	Pulmonary NC	- Baseline [18F]FDG PET/CT	Left upper lobe lesion (SUVmax 22.2)	- Primary: left upper lobe lesion (SUVmax 22.2). - Metastases: mediastinal lymph nodes (SUVmax 14.5), left laryngeal nerve involvement	NR	PET showed heterogeneous FDG uptake in the primary pulmonary lesion
Jimenez et al., 2023 [28]	5 y, F	Head and neck NC	- Baseline [18F]FDG PET/CT	Left mandibular mass (SUVmax 9.4)	- Primary: left mandibular mass with adjacent soft-tissue extension. - Metastases: none	Remission 21 months after the end of therapy	PET/CT documented disease extent and peripheral FDG uptake around a necrotic center in the primary mandibular lesion
Liu et al., 2025 [8]	38 y, F	Pulmonary NC	- Staging [68Ga]Ga-FAP-2286 PET/CT	Right hilar lung mass (SUVmax 10.9)	- Primary: right lung hilum mass (SUVmax 10.9) - Metastases: left mediastinal lymph nodes (SUVmax 9.6), L5 vertebra, left adnexa with a soft tissue mass (SUVmax 12.9), sacrum and left iliac bone (SUVmax 15.8)	NR	[68Ga]Ga-FAP-2286 PET/CT showed uptake in the pulmonary primary and multiple metastatic sites, although evidence remains anecdotal
Schlemmer et al., 2025 [16]	32 y, M	Pulmonary NC	- Baseline [18F]FDG PET/CT	Right lung mass (SUVmax 14)	- Primary: right lung mass (SUVmax 14) - Metastases: subcarinal lymph node conglomerate (SUVmax 11.3)	NR	PET showed peripheral FDG uptake around central tumor necrosis, illustrating heterogeneous viable tumor uptake within necrotic masses
Zhang et al., 2025 [35]	38 y, F	Pulmonary NC	- Restaging [68Ga]Ga-DOTA-IBA PET/CT	Right lung mass (SUVmax 6.6)	- Primary: right lung (SUVmax 6.6). - Metastases: bone metastases; subcutaneous soft tissue nodule in the left lower back region (SUVmax 3.8)	NR	[68Ga]Ga-DOTA-IBA showed uptake in skeletal lesions as well as the primary lung lesion and an extraosseous subcutaneous nodule; any theranostic implication remains unproven
Schulz et al., 2026 [7]	19 y, M	Head and neck NC	- Baseline [18F]FDG PET/CT - After 2 cycles of chemoradiotherapy-guided PET/CT: no abnormal FDG-avid disease. - 5-year follow-up PET/CT: no abnormal FDG-avid disease	Supraglottic primary tumor (SUVmax NR)	- Primary: supraglottic tumor with extension to the hypopharynx and tongue base - Metastases: bilateral cervical lymph nodes	Complete remission beyond 7 years	This long-term survivor had early disappearance of abnormal FDG uptake after therapy; PET/CT was also used in radiotherapy planning
Xiao et al., 2026 [24]	23 y, M	Pulmonary NC	- Baseline [18F]FDG PET/CT	Right distal little finger metastasis (SUVmax 12.5)	- Primary: right lung - Metastases: right mediastinal/supraclavicular/axillary lymph nodes, bilateral adrenal lesions, multiple bone metastases	NR	PET/CT identified non-osteoblastic skeletal metastases not demonstrated on bone scintigraphy, including unusual sites such as the distal phalanx, mandibular condyle, and thyroid cartilage
Zhang et al., 2026 [33]	10 y, F	Right kidney NC	- Staging [68Ga]Ga-FAPI-04 PET/CT	Right pulmonary metastases (SUVmax 13.94)	- Primary: right renal mass - Metastases: retroperitoneal lymphadenopathies (SUVmax 10.85), right pulmonary metastases (SUVmax 13.94)	NR	[68Ga]Ga-FAPI-04 PET/CT showed uptake in the renal primary NC and metastatic sites
Wang et al., 2026 [34]	31 y, M	Mediastinal NC	- Baseline [18F]FDG and [68Ga]Ga-FAPI-04 PET/CT comparison	Mediastinal mass (SUVmax 34.0) on [18F]FDG PET; pleural lesions on [68Ga]Ga-FAPI-04 PET/CT (SUVmax 9.1)	[18F]FDG PET/CT: - Primary: anterior mediastinal mass (SUVmax 34.0). - Metastases: left subclavian lymph node (SUVmax 6.2). - [68Ga]Ga-FAPI-04 PET/CT: - Primary: mediastinal mass (SUVmax 8.6). - Metastases: pleural lesions (SUVmax 9.1)	NR	[18F]FDG showed higher uptake in the primary tumor and nodal disease, whereas [68Ga]Ga-FAPI-04 delineated pleural lesions more clearly

Abbreviations: CHT, chemotherapy; CT, computed tomography; [18F]FDG PET/CT, 2-deoxy-2-[18F]fluoro-D-glucose positron emission tomography/computed tomography; F, female; FAP, fibroblast activation protein; FAPI, fibroblast activation protein inhibitor; M, male; MRI, magnetic resonance imaging; NC, NUT carcinoma; NR, not reported; OS, overall survival; RT, radiotherapy; SUVmax, maximum standardized uptake value; y, years. * The Virarkar et al. publication is displayed in two site-specific rows according to the primary site.

## 4. Conclusions

[18F]FDG PET/CT may provide information complementary to CT and MRI in NC. At baseline, whole-body metabolic assessment with [18F]FDG PET/CT may complement disease staging by identifying skeletal lesions without CT correlates or by prompting additional MRI evaluation; it may also help characterize metabolically active tissue within large heterogeneous or necrotic masses, potentially supporting biopsy targeting.

Serial [18F]FDG PET/CT was used in individual cases to document treatment-related metabolic changes and heterogeneous progression patterns.

FDG uptake in NC is highly heterogeneous, and both SUVmax and PET-derived volumetric parameters should be interpreted as descriptive PET measurements in the individual clinical context. MTV and TLG remain exploratory variables for future investigation.

Overall, this three-case series and structured narrative review illustrate the complementary information that [18F]FDG PET/CT may provide within multimodality imaging of NC. Larger multicenter studies using standardized PET acquisition, segmentation, analysis, and longitudinal interpretation methods are needed to define the optimal timing of PET/CT and to determine whether PET-derived quantitative measures provide clinically meaningful information beyond established clinical prognostic factors.

## Figures and Tables

**Table 1:** Clinical and [18F]FDG PET/CT findings of the three patients with NUT carcinoma.

Feature	Case #1	Case #2	Case #3
**Age/sex**	13 y, M	20 y, F	8 y, M
**Primary site**	Anterior chest wall	Head and neck region	Mediastinal region
**PET/CT timing and findings**	**Baseline** **Post-therapy**: 1 month after the end of the 11th CHT cycle: marked decrease in FDG uptake in the chest wall region **Follow-up**: 5 months after the end of therapy: no FDG-avid findings suggestive of recurrence	**Baseline** **Post-therapy**: 15 days after the 4th CHT cycle: marked decrease in FDG uptake in the primary site; no residual FDG uptake at the previously involved nodal and skeletal sites **Follow-up**: 3 months after the end of therapy: increased locoregional FDG uptake with nodal and mandibular involvement	**Baseline** **Interim**: after two cycles of CHT: marked decrease in FDG uptake at the main involved sites **Post-therapy**: 17 days after the end of therapy: persistent reduction in thoracic uptake with multiple FDG-avid skeletal lesions
**Baseline PET semiquantitative analysis: SUVmax**	Primary lesion: 7.2	- Primary lesion: 29.1 - Cervical lymph nodes: 22.9 - Bone lesions: 14.5 at L3	- Primary lesion: 12.4 - Mediastinal lymph nodes: 12.2 - Bone lesions: 16.3
**MTV**	Primary lesion: 126 cm^3^	Primary lesion: 416 cm^3^ Whole-body: 424.364 cm^3^	Primary lesion: 114 cm^3^ Whole-body: 152.17 cm^3^
**TLG**	Primary lesion: 580.57 g/mL × cm^3^	Primary lesion: 3987.90 g/mL × cm^3^ Whole-body: 4045.87 g/mL × cm^3^	Primary lesion: 832.74 g/mL × cm^3^ Whole-body: 1224.6 g/mL × cm^3^
**Outcome**	Alive at 30-month follow-up	Died OS: 11 months	Died OS: 12 months
**Case-level PET/CT observations**	In this localized, R0-resected case, serial [18F]FDG PET/CT demonstrated post-treatment metabolic changes, with no FDG-avid findings suggestive of recurrence during follow-up. For descriptive completeness, this patient had the lowest baseline SUVmax and PET-derived volumetric tumor burden among the three cases; however, no relationship with outcome can be inferred because baseline stage, resectability, and treatment intent differed substantially across patients.	Baseline [18F]FDG PET/CT identified FDG-avid skeletal lesions without corresponding structural abnormalities on CT. After therapy, disappearance of abnormal FDG uptake at these sites helped contextualize subsequently visible bone remodeling on CT. This individual observation illustrates the complementary value of multimodality imaging.	Interim [18F]FDG PET/CT documented an early decrease in FDG uptake in the main involved sites, followed by the appearance of new bone lesions despite persistent reduction in thoracic FDG uptake on the post-treatment examination. Imaging time points were clinically driven and non-standardized.

Abbreviations: M, male; F, female; y, years; CHT, chemotherapy; SUVmax, maximum standardized uptake value; OS, overall survival; PET/CT, positron emission tomography/computed tomography; FDG, fluorodeoxyglucose; CT, computed tomography; MTV, metabolic tumor volume; TLG, total lesion glycolysis.

## Data Availability

The data that support the findings of this study are available on request from the corresponding author. The data are not publicly available due to privacy and ethical restrictions.

## Abbreviations

The following abbreviations are used in this manuscript:

NC	NUT carcinoma
RT	Radiotherapy
CHT	Chemotherapy
PET	Positron Emission Tomography
OS	Overall Survival
MTV	Metabolic Tumor Volume
TLG	Total Lesion Glycolysis
